# Influence of different kinds of surgical resection on operation-related clinical indexes, inflammatory cytokines and complications in elderly patients with esophageal cancer

**DOI:** 10.12669/pjms.36.3.1465

**Published:** 2020

**Authors:** Tao Zhang, Hai Jiang, Chen Yu Ming, Yan Wang, Deng Mao, Yuan fu Wei

**Affiliations:** 1 Tao Zhang, Department of Cardiothoracic Vascular Surgery, Renmin Hospital, Hubei University of Medicine, Shiyan, Hubei, China, P.R. 442000; 2 Hai Jiang, Department of Cardiothoracic Vascular Surgery, Renmin Hospital, Hubei University of Medicine, Shiyan, Hubei, China, P.R. 442000; 3 Chen Yu Ming, Department of Cardiothoracic Vascular Surgery, Renmin Hospital, Hubei University of Medicine, Shiyan, Hubei, China, P.R. 442000; 4 Yan Wang, Department of Cardiothoracic Vascular Surgery, Renmin Hospital, Hubei University of Medicine, Shiyan, Hubei, China, P.R. 442000; 5 Deng Mao, Department of Cardiothoracic Vascular Surgery, Renmin Hospital, Hubei University of Medicine, Shiyan, Hubei, China, P.R. 442000; 6 Yuan fu Wei, Department of Cardiothoracic Vascular Surgery, Renmin Hospital, Hubei University of Medicine, Shiyan, Hubei, China, P.R. 442000

**Keywords:** Esophageal cancer, Laparoscopy, Open, Thoracoscopy

## Abstract

**Objective:**

To investigate the effects of two kinds of surgical resection schemes, a conventional open surgical scheme and a thoracolaparoscopic esophagectomy surgical scheme, on operation-related clinical indexes, inflammatory cytokines and complications in elderly patients with esophageal cancer.

**Methods:**

A total of 100 elderly patients with esophageal cancer seen in the Department of Cardiothoracic Vascular Surgery, Renmin Hospital, Hubei University of Medicine, from June 2014 to June 2016 were enrolled and randomly divided into two groups, including a control group (50 patients) with a conventional open surgical scheme and an observation group (50 patients) with a thoracolaparoscopic esophagectomy surgical scheme. The operation time, the amount of bleeding during the operation, the incision length, the number of lymph nodes dissected, the hospitalization time, the HAMA scores and HAMD scores before and after the operation, the PSQI scores, SF-36 scores and levels of PCT, CRP and IL-6 after the operation, the recurrence and metastasis rates and the mortality at follow-up and the incidence of related complications of both groups were compared.

**Results:**

The operation time, the amount of bleeding during the operation, the incision length and the hospitalization time in the observation group were significantly less than those in the control group (p<0.05). The number of lymph nodes dissected in the observation group was significantly higher than that in the control group (p<0.05). The HAMA scores and HAMD scores after the operation in the observation group were significantly lower than those in the control group and those before the operation (p<0.05). The PSQI scores and SF-36 scores after the operation in the observation group were significantly better than those in the control group and those before the operation (p<0.05). The levels of PCT, CRP and IL-6 after the operation in the observation group were significantly lower than those in the control group (p<0.05). The recurrence and metastasis rates at follow-up in the observation group were significantly lower than those in the control group (p<0.05). There was no significant difference in mortality at follow-up between the two groups (p>0.05). The complication incidence after the operation in the observation group was significantly lower than that in the control group (p<0.05).

**Conclusion:**

Compared with a conventional open surgical scheme, the thoracolaparoscopic esophagectomy surgical scheme possesses advantages in the treatment of elderly patients with esophageal cancer, including being a minimally invasive, simple operation, having a shorter recovery time, effectively relieving negative emotions, improving the quality of life, reducing the levels of inflammatory molecules and reducing the risk of related complications.

## INTRODUCTION

Esophageal cancer is one of the most common malignant tumors of the digestive system and ranks 7^th^ among malignant tumors with respect to morbidity and mortality worldwide. The number of elderly patients with esophageal cancer accounts for 70% to 85% of the total number of patients with esophageal cancer.^1^ The main clinical symptom of patients with this disease is progressive dysphagia, with malnutrition and immune abnormalities, which greatly lowers the quality of life of patients.^2^ The first choice for clinical treatment of patients with esophageal cancer is surgical resection, but clinical reports show that an open surgical scheme is likely to cause severe iatrogenic trauma in patients, and postoperative intense pain and a local, excessive inflammatory response may substantially lengthen the time for recovery, which adversely affects long-term prognosis.^3^

In recent years, as endoscopic techniques have advanced and surgeons’ operational proficiencies continue to improve, a mini-invasive surgical treatment scheme represented by thoracolaparoscopic esophagectomy has been widely applied for the clinical treatment of patients with esophageal cancer,^4^ but whether the thoracolaparoscopic esophagectomy surgical scheme is superior to an open surgical scheme with respect to curative effects and safety remains controversial. For the purpose of this study, 100 elderly patients with esophageal cancer were treated with an open surgical scheme or a thoracolaparoscopic esophagectomy surgical scheme to study the influence of different kinds of surgical resection on operation-related clinical indexes, inflammatory cytokines and complications in this patient population.

## METHODS

One hundred elderly patients with esophageal cancer hospitalized in Renmin Hospital, Hubei University of Medicine, from June 2014 to June 2016 were divided into two equal groups (50 patients per group) with a random number table, namely, a control group and an observation group. The control group consisted of 32 male patients and 18 female patients, 37 cases of squamous carcinoma and 13 cases of adenocarcinoma according to histopathological typing, and 3 cases of upper thoracic esophageal carcinoma, 37 cases of middle thoracic esophageal carcinoma, and 10 lower thoracic esophageal carcinoma according to tumor location, with ages ranging from 65 to 78 years (average age 71.32±5.66). The observation group consisted of 32 male patients and 18 female patients, 37 cases of squamous carcinoma and 13 cases of adenocarcinoma according to histopathological typing, and three cases of upper thoracic esophageal carcinoma, 37 cases of middle thoracic esophageal carcinoma, and 10 lower thoracic esophageal carcinoma according to tumor location, with ages ranging from 65 to 78 years (average age 71.32±5.66). There were no significant differences between the two groups in terms of these general characteristics (*p*>0.05).

The study was approved by the Institutional Ethics Committee of Renmin Hospital, Hubei University of Medicine dated July 23, 2019 and written informed consent was obtained from all participants.

### Inclusion Criteria:

1. Patients that had been diagnosed with esophageal cancer via esophagus barium meal examination and gastroendoscopic biopsy.
2. Patients with tumor foci in thoracic segments.
3. Patients with age≥65.
4. Patients with a KPS score>60.
5. Patients for which the treatment schedule had been approved by the hospital ethics committee and whose family members had signed the informed consent form.

### Exclusion Criteria:

1. Patients with disease involvement in nearby lymph nodes or distant metastasis
2. Patients with pleural adhesions that were difficult to separate.
3. Patients with malignant tumor of other systems.
4. Patients with circulatory system diseases.
5. Patients with hematological system diseases.
6. Patients with narcotic contraindications.
7. Patients with immune system diseases.
8. Patients with heart, brain, and hepatic or renal dysfunction.
9. Patients with insufficient clinical data.

The patients of both groups received double-lumen tube intubation under general anesthesia. Patients in the control group underwent an open surgical scheme, including resection of the posterolateral right thorax, center of the superior stomach, and front side of the left sternocleidomastoid, excision of the tumor, and three-field dissection of lymph nodes, and the gastric tube was lifted to the neck to complete the anastomosis. Patients in the observation group received a thoracolaparoscopic esophagectomy surgical scheme; patients were put in left lateral position, a 1.5-2.0 cm incision was made at the seventh intercostal in the midaxillary line, and the mediastinum lymph nodes were first probed by means of a thoracoscope. Next, 1.0 cm incisions were made at the 6^th^ and 9^th^ intercostals in the infrascapular line and at the 3^rd^ intercostal in the anterior axillary line, and the esophagus was separated at thoracic segments. Patients were then put in a horizontal position, a 5-6 cm incision was made at the center of the superior stomach. The stomach separation was completed by means of a thoracoscope, resulting in division of the esophagus at the gastric cardia. Finally, a 4-5 cm incision was made at the left sternocleidomastoid, and the anastomosis between the esophagus and gastric tube was completed).

### Observation Target:

1. Record the time taken for surgery, intraoperative bleeding volume, length of the incision, number of dissected lymph nodes, and total length of hospital stay, and calculate the respective percentages
2. Evaluate the emotional status of the patients with HAMA scores and HAMD scores (the scores are positively related to the severity of depression and anxiety).^5^
3. Evaluate the quality of life in patients with PSQI scores and SF-36 scores (the PSQI score is negatively related to sleep quality, while the SF-36 score is positively related to overall quality of life)^5^
4. Detect inflammatory cytokine indexes including PCT, CRP, and IL-6 with a Roche Cobas c311 automatic biochemical analyzer.
5. Perform a 12-month follow-up visit to record the number of patients who have had recurrence or metastasis and the number of patients who have died and calculate the respective percentages.
6. Record the incidence of postoperative fat liquefaction, pulmonary infection, hoarseness, anastomotic fistula and arrhythmia and calculate the respective percentages.
7. ***Statistical analysis:*** SPSS 20.0 software was employed for data analysis; for categorical data, Student’s t-test was employed, and results were reported as the mean ± standard deviation; for numerical data, the χ2 test was employed, and results were reported as percentages (%); α=0.05 was set as the significance cutoff.

## RESULTS

The time taken for surgery, intraoperative bleeding volume, length of the incision, and total length of hospital stay in the observation group were significantly less than those in the control group (*p*<0.05); the number of dissected lymph nodes in the patients in the observation group was significantly more than that in the patients in the control group (*p*<0.05); Table-I.

**Table-I:** Comparison of time taken for surgery, intraoperative bleeding volume and total length of hospital stay between the two groups.

Group	Count of case	Time taken for surgery	Intraoperative bleeding volume	Length of incision (cm)	Lymph node biopsy and count	Total length of hospital stay
Control group	50	372.74±55.14	517.59±79.28	18.59±3.28	31.67±3.15	11.41±3.08
Observation control	50	307.30±41.28※	231.33±44.71※	6.13±1.05※	42.50±5.84※	7.75±1.52※

※ By comparing with the control group, p<0.05.

The time taken for surgery, intraoperative bleeding volume, length of the incision, and total length of hospital stay in the observation group were significantly less than those in the control group (*p*<0.05); the number of dissected lymph nodes in the patients in the observation group was significantly more than that in the patients in the control group (*p*<0.05); Table-II.

**Table-II:** Comparison of Emotional Status before and after Operation between the Two Groups (Score).

Group	Count of case	HAMA score		HAMD score	

		Before	After	Before	After
Control group	50	25.95±4.60	19.26±2.81Δ	22.16±4.70	15.85±3.97Δ
Observation group	50	25.70±4.56	13.07±1.64Δ※	21.80±4.63	12.23±2.48Δ※

※ By comparing with the control group, p<0.05; Δ by comparing with itself before treatment, p<0.05.

### Comparison of postoperative quality of Life between the two groups

The postoperative PSQI score of the patients in the observation group was significantly lower than that of the patients in the control group (*p*<0.05). The postoperative SF-36 score of the patients in the observation group was significantly higher than that of the patients in the control group (*p*<0.05. Table-III.

**Table-III:** Comparison of Living Quality between the Two Groups.

Group	Count of case	PSQI score	SF-36 score
Control group	50	8.19±1.77	70.53±7.17
Observation group	50	4.60±0.95※	87.69±10.80※

※ By comparing with the control group, p<0.05.

The postoperative PCT, CRP and IL-6 levels of the patients in the observation group were significantly lower than those of the patients in the control group (*p*<0.05). Table-IV.

**Table-IV:** Comparison of Postoperative Inflammatory Response Level between the Two Groups.

Group	Count of case	PCT (ug/L)	CRP (mg/L)	IL-6 (ng/L)
Control group	50	5.61±1.25	116.71±23.16	283.72±52.94
Observation group	50	1.47±0.34※	54.37±10.82※	220.54±39.10※

※ By comparing with the control group, p<0.05.

During the 1-year follow-up period, the rate of recurrence and metastasis of the patients in the observation group was significantly lower than that of the patients in the control group (*p*<0.05); there was no significant difference between the two groups in terms of survival (*p*>0.05). Table-V.

**Table-V:** Comparison of Rate of Recurrence and Metastasis and Survival Rate between the Two Groups [n, %].

Group	Count of case	Rate of recurrence and metastasis	Survival rate
Control group	50	12 (24.00)	48 (96.00)
Observation group	50	5 (10.00)	49 (98.00)

※ By comparing with the control group, p<0.05.

The incidence of complications in the patients in the observation group was significantly lower than that in the patients in the control group (*p*<0.05). Table-VI.

**Table-VI:** Comparison of Incidence of Complications between the Two Groups.

Group	Count of case	Fat liquefaction	Pulmonary infection	Hoarseness	Anastomotic fistula	Arrhythmia	Incidence of complications (%)
Control group	50	2	7	2	2	1	28.00
Observation group	50	0	1	0	1	1	6.00※

※ By comparing with the control group, p<0.05.

## DISCUSSION

More than two hundred thousand new patients were recently diagnosed with esophageal cancer in China, and this incidence has increased over the years, accounting for 25% to 35% of all new patients worldwide.^6^ To date, the pathogenesis of esophageal cancer has not been explicitly defined, and excessive intake of hot and spicy foods, excessive intake of nitrite-containing foods, smoking, excessive drinking, hypovitaminosis, and genetic factors are considered to be related to the occurrence and development of the disease.^7^ Patients with esophageal cancer should receive surgical resection as often as possible, such as open surgical schemes, small incision surgical schemes, total thoracoscopy surgical schemes, and thoracolaparoscopic esophagectomy surgical schemes.^8^ In recent years, the thoracolaparoscopic esophagectomy surgical scheme has been gradually recognized in the medical field for its advantages, namely, minimal invasiveness, a satisfactory level of lymph node dissection and a short postoperative recovery time,^9^ but there is no clear conclusion about whether the thoracolaparoscopic esophagectomy surgical scheme can improve the overall clinical benefit in patients.

According to the results of this research, the time taken for surgery, intraoperative bleeding volume, length of the incision, and total length of hospital stay of the patients in the observation group were significantly less than those of the patients in the control group (*p*<0.05); the number of dissected lymph nodes in the patients in the observation group was significantly more than that in the patients in the control group (*p*<0.05), indicating that the thoracolaparoscopic esophagectomy surgical scheme largely reduced the operative wound and operative difficulty, promoted postoperative rehabilitation, and improved the effect of lymph node dissection.^10^ The main reasons for the reduction in the time taken for surgery and intraoperative bleeding volume that resulted from adopting the surgical scheme include

1. The operation of surgical instruments with the aid of an endoscopic tube is more smooth
2. It is difficult to carry out the open surgical scheme in the gastric tube.
3. Bleeding can be stopped quickly in the case of electrocautery

The postoperative HAMA score, HAMD score, PSQI score and SF-36 score of the patients in the observation group were significantly better than those of the patients in the control group and those before treatment (*p*<0.05), indicating that treatment of elderly patients with esophageal cancer with the thoracolaparoscopic esophagectomy surgical scheme is helpful for relieving negative emotions in patients and improving patient sleep and overall quality of life, and the above mentioned advantages of the thoracolaparoscopic esophagectomy surgical scheme are closely related to the alleviation of the operative wound and postoperative pain and the reduction in recovery time.

The postoperative PCT, CRP and IL-6 levels of the patients in the observation group were significantly lower than those of the patients in the control group (*p*<0.05), suggesting that the thoracolaparoscopic esophagectomy surgical scheme is significantly superior with respect to reducing the systemic inflammatory response and preventing postoperative infection during the treatment of elderly patients with esophageal cancer.^11^ PCT, CRP and IL-6 are common inflammatory cytokine indexes that reflect the body’s immune response and stress response, and all of these markers continually increase in the case of infection.^12^

During the one year follow-up period, the rate of recurrence and metastasis in the patients in the observation group was significantly lower than that in the patients in the control group (*p*<0.05). The incidence of complications in the patients in the observation group was significantly lower than that in the patients in the control group (*p*<0.05), indicating that the thoracolaparoscopic esophagectomy surgical scheme is helpful for further reducing the risk of long-term recurrence and metastasis and preventing the occurrence of complications after operation in the treatment of elderly patients with esophageal cancer. The thoracolaparoscopic esophagectomy surgical scheme is helpful for ensuring normal tension in surrounding tissue during separation of the base of the esophagus and increases the anatomic space exposed, which is of great significance for preventing accidental nerve injury and secondary hoarseness.^13^,^14^ The reduction in the postoperative systemic inflammatory cytokine level of patients treated with the thoracolaparoscopic esophagectomy surgical scheme relative to patients treated with the open surgical scheme is considered to be an important reason for the reduction in the incidence of pulmonary infection.^15^ However, the comparison of survival during the one year follow-up period between the two groups found no significant difference (*p*>0.05), which we believe to be related to the insufficient number of samples and the short follow-up time. As such, these findings need to be further verified in a controlled study on a larger scale.

## CONCLUSION

In summary, the thoracolaparoscopic esophagectomy surgical scheme has the advantages of minimal invasiveness, ease of operation, and short postoperative recovery time and is superior to the open surgical scheme with respect to relieving negative emotions in patients, improving patient quality of life, reducing the inflammatory response, and helping reduce the risk of complications.

### Authors’ Contributions:

**TZ** **and HJ** designed this study, prepared this manuscript, and are responsible and accountable for the accuracy of the work.

**YW and CYM** collected and analyzed clinical data.

**YW and** **DM** significantly revised this manuscript.

**TZ and HJ** contributed equally to this manuscript.
